# Changes in Macular Pigment Optical Density and Serum Lutein Concentration in Japanese Subjects Taking Two Different Lutein Supplements

**DOI:** 10.1371/journal.pone.0139257

**Published:** 2015-10-09

**Authors:** Akira Obana, Masaki Tanito, Yuko Gohto, Shigetoshi Okazaki, Werner Gellermann, Paul S. Bernstein

**Affiliations:** 1 Department of Ophthalmology, Seirei Hamamatsu General Hospital, Hamamatsu, Shizuoka, Japan; 2 Department of Medical Spectroscopy, Applied Medical Photonics Laboratory, Medical Photonics Research Center, Hamamatsu University School of Medicine, Hamamatsu, Shizuoka, Japan; 3 Department of Ophthalmology, Shimane University Faculty of Medicine, Izumo, Shimane, Japan; 4 Department of Physics and Astronomy, University of Utah, Salt Lake City, Utah, United States of America; 5 Department of Ophthalmology and Visual Sciences, Moran Eye Center, University of Utah School of Medicine, Salt Lake City, Utah, United States of America; University of Florence, ITALY

## Abstract

**Purpose:**

To investigate macular pigment optical density (MPOD) and serum concentration changes of lutein in Japanese subjects participating in a clinical trial in which two formulations of lutein and zeaxanthin supplements with different physiochemical properties are used.

**Methods:**

Thirty-six healthy volunteers were recruited into this prospective, randomized, parallel-group, double-masked comparative study at a single institute. Two products were used, FloraGLO® (Kemin Japan) and XanMax® (Katra Phytochem). The lutein particle size and zeaxanthin concentrations differed between the formulations. The subjects consumed one of the two supplements for a duration of up to 6 months. MPOD levels were measured by resonance Raman spectrometry at baseline and once a month until the end of the study. Serum lutein concentration was measured at baseline, month 3, and month 6. The subjects were also tested for contrast sensitivity, glare sensitivity, visual acuity, and in addition had a focal electroretinogram measured.

**Results:**

The mean serum lutein concentrations increased significantly after the first three months, but the mean MPOD levels in either supplement group did not show any statistically significant increase. A detailed analysis, however, revealed three response patterns in both groups for the increase of MPOD levels and serum lutein concentration, i.e. “retinal responders”, who had an increase of both MPOD levels and serum lutein concentrations (n = 13), “retinal non-responders”, who had only increased serum concentrations and no change in MPOD levels (n = 20), and “retinal and serum non-responders”, who had neither MPOD level nor plasma concentration increases (n = 3). The subjects with low MPOD levels at baseline appeared to show increased MPOD levels at the 6 month time point upon lutein supplementation (r = -0.4090, *p* = 0.0133). Glare sensitivity improved in retinal responders in both supplement groups, while there were no remarkable changes in contrast sensitivity.

**Conclusions:**

No statistically significant differences could be detected for MPOD levels and serum lutein concentrations between the two investigated lutein supplement formulations. Responses to lutein supplementation regarding MPOD levels and serum lutein concentrations varied between subjects. Subjects with lower MPOD levels at baseline responded well to lutein supplementation. However, since the number of subjects was low, a further study with more subjects is needed to prove that subjects with low MPOD levels will benefit from lutein supplementation.

**Trial Registration:**

UMIN-CTR UMIN000004593

## Introduction

The yellow human macular pigment consists of three carotenoids, lutein ((3R,3’R,6’R)-lutein), zeaxanthin ((3R,3’R)-zeaxanthin), and *meso*-zeaxanthin ((3R,3’S;*meso*)-zeaxanthin) [1,2]. It absorbs blue light and acts as a filter that might attenuate photochemical damage of the retina from blue light exposure. It also works as an antioxidant that may protect against light-induced oxidative damage in the retina via quenching of oxygen radicals [3,4]. These light protection effects of macular pigment help prevent age-related macular degeneration (AMD), a major cause of legal blindness in aged people [5–9]. A multi-center, randomized trial investigating the progression from an early stage of age-related maculopathy to advanced AMD, has revealed a prophylactic effect of lutein- and zeaxanthin-containing anti-oxidative supplements, at least for the quintile with the lowest dietary intake of lutein and zeaxanthin [10].

Humans are not able to synthesize lutein and zeaxanthin, so we have to obtain them from dietary sources such as green leafy vegetables or from supplements. The hydrophobic lutein and zeaxanthin carotenoids are absorbed into the small intestine in micellarized forms [11], and their bioavailability is affected by many factors such as gut health, genotype, and dietary lipid components taken in combination with these carotenoids. In addition, the physiochemical properties of lutein crystals, such as their size, play a role in the bioavailability. Generally, smaller lutein particles are thought to dissolve into lipids and to micellarize more efficiently than larger size particles, and also to absorb more efficiently than larger size particles [12].

In the current study, we investigated the efficacy to increase MPOD levels and serum lutein concentrations in normal, healthy Japanese subjects using two lutein supplement formulations; i.e. FloraGLO® lutein (Kemin Japan, Tokyo, Japan), which is the most widely used lutein supplement globally, and XanMax® lutein (Katra Phytochem, Bangalore, India), which features smaller particle sizes compared to FloraGLO.

## Subjects and Methods

### Supplement

FloraGLO and XanMax supplements both contain lutein obtained from marigold oleoresin, but they differ in their oleoresin extraction method from marigold flowers (*Tagetes erecta*) and in their lutein crystallization method. Both products contain the free (unesterified) form of lutein, but the particle size of lutein is different. We measured the exact amount of lutein and zeaxanthin in each product used in the present study by HPLC. The contents of each product are shown in Table 1.

**Table 1:** Contents of supplements tested in the present study.

	FloraGLO (Kemin Japan)	XanMax (Katra Phytochem)
Weight of total contents in one capsule (mg)	199	202
Lutein contained in one capsule (mg)	10.5	10.4
Size of lutein particle (μm)	9	4
Zeaxanthin contained in one capsule (mg)	0.96	1.29
The ratio of lutein: zeaxanthin	10: 0.9	10: 1.2
Suspension	Corn oil	Safflower oil

### Subjects

**Fig 1:**
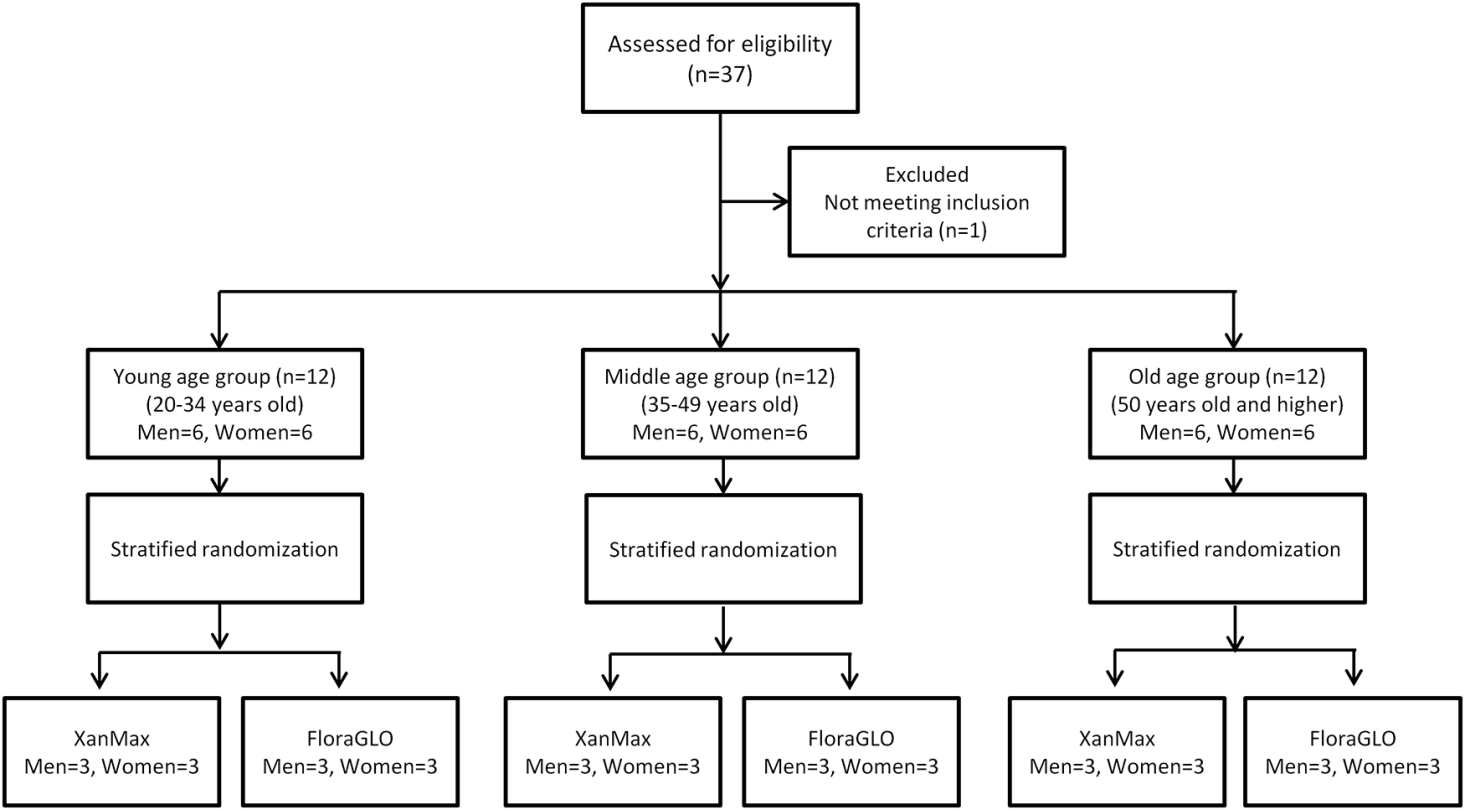
Flow diagram of the study.

This is a prospective, parallel-group comparison, double-masked study at a single institute (Seirei Hamamatsu General Hospital) (S1 and S2 Protocols, S1 CONSORT Checklist). We recruited healthy volunteers from December 2010 to December 2011. We performed a stratified randomization and enrolled three age groups: group 1 ranged from 20 to 34 years old, group 2 ranged from 35 to 49 years old, and group 3 was 50 years old and higher (Fig 1). Each age group had 12 subjects (6 men and 6 women), and they were assigned by a computer-generated table of random numbers to one of two groups, i.e.FloraGLO or Xanmax. Each group contained 3 men and 3 women taking FloraGLO, and 3 men and 3 women taking XanMax, respectively. All subjects were healthy Japanese without ocular or systemic pathologies who had no history of taking lutein, zeaxanthin, or any vitamin supplements. Each subject took one FloraGLO or XanMax capsule orally daily for 6 months. The body mass index (BMI), defined as the body weight in kilograms divided by the square of the height in meters, was determined based on the patient body weight and height at baseline (1 day before the start of supplementation). Details of the inclusion criteria are shown in Table 2. The study was approved by the institutional review board of Seirei Hamamatsu General Hospital (No.854). All subjects signed an informed consent form that complied with the tenets of the Declaration of Helsinki. (UMIN000004593)

**Table 2:** Inclusion Criteria.

Japanese (Asian)
No ocular pathologies detected by slit-lamp biomicroscopy and fundus ophthalmoscopy
Visual acuity of 0.8* or better at the time of the MPOD measurement
A spherical equivalent refractive error of less than -6.0 diopter
No gastrointestinal diseases that could cause disturbance of dietary absorption
No diabetes
No history of lutein supplementation
No allergy to lutein and zeaxanthin
No history of smoking at least within one year
Pupil diameter of 7.0 mm or more at the time of MPOD measurement

* The visual acuity was measured using a decimal visual acuity test chart. 0.8 was equivalent to 20/25 of Snellen visual acuity or 0.097 of logMAR.

MPOD = macular pigment optical density.

### Assessment of Ocular Conditions

Measurements of visual acuity and intraocular pressure and observation with slit-lamp biomicroscopy and fundus ophthalmoscopy were performed prior to subject recruitment in order to assess the inclusion criteria. Also, fundus color photographs were taken at that time. Subjects underwent visual acuity measurements and contrast and glare sensitivity testing, using a contrast glare-tester (Model CGT-1000, TAKAGI, Nagano, Japan), at baseline, and at 3 months and 6 months after the start of supplementation. With the CGT-1000, contrast threshold values were assessed at six visual angles (sizes) of the target (6.3, 4.0, 2.5, 1.6, 1.0, 0.7 degrees) under mesopic (10 candelas per square meter) conditions. The thresholds were also assessed under glare (10,000 candelas per square meter) conditions using the same target sizes.

The study eye was determined based on subject preference because both eyes of all subjects met the inclusion criteria; only the study eye underwent the following examinations. For functional assessment, the retinal function of the central fovea was examined using focal electroretinography (Visual Stimulator ER-80, Kowa, Aichi, Japan) at baseline and at 6 months after the start of supplementation.

### Macular Pigment Optical Density (MPOD) Measurement

MPOD levels were measured in the study eye using resonance Raman spectrophotometry (RRS) at baseline and every month until the end of this study, which was 6 months after the start of supplementation. Two trained technicians who were masked to the groups performed all MPOD measurements. The RRS device and measurement procedures were described previously [13,14]. In the current study, the RRS module was interfaced with the front end optics of a commercial wide-angle fundus camera (Carl Zeiss, Oberkochen, Germany). This system allows the operator to quickly locate and center the macula for macular pigment Raman measurements. Prior to measurements the pupil was dilated to at least 7 mm diameter using a topical mydriatic agent. To eliminate artifactually low RRS intensities due to ocular misalignments or blinking, measurements were performed five times in each visit, and the maximal RRS value was used for data analysis.

### Measurement of Serum Lutein Concentration

Blood samples were taken from each subject at baseline, 3 months, and 6 months after the start of supplementation. Serum lutein analysis was conducted by the Diagnostic Division of Otsuka Pharmaceutical Co., Ltd. (Tokushima, Japan). For this purpose, 0.5 mL of serum was mixed with 25μL of ethyl 8’-apo-beta-caroten-8’-oate (100 μg/mL in *n*-hexane [internal standard]), 1.25 mL ethanol containing 0.01%BHT, and 0.75 mL distilled water. After mixing for 1 minute, 5 mL of *n*-hexane was added to the sample and mixed for 2 minutes. The sample was centrifuged (1500 rpm, 5 min.), and *n*-hexane extracts were evaporated under N_2_ gas. Finally, the residue was dissolved in 250μL of acetonitrile: methanol: chloroform = 60: 25: 15, and the solution was centrifuged (1500 rpm, 5min). The supernatant was injected onto the HPLC system (Model 5600A CoulArray Detector, Thermo Scientific, CA).

### Statistical Analysis

Statistical analyses were performed on a Macintosh personal computer using StatView software (version 5.0, SAS Institute, Inc., Cary, NC) or JMP software (version 8.0, SAS Institute, Inc.). All statistical tests were two-sided; *P* < 0.05 was considered significant. For comparisons between FloraGLO and XanMax groups, age and spherical equivalent refractive error were compared by un-paired t- tests; gender difference was compared by Fisher’s exact probability test. Changes of MPOD levels during the trial phases were analyzed with a multivariate approach, specifically with a multivariate analysis of variance (MANOVA). This method has an advantage over a univariate approach with split-plot and repeated-measures analysis of variance, since it does not require a sphericity assumption.

When analyzing changes of MPOD levels and serum lutein concentrations, we found certain patterns between the change of MPOD levels and change of serum lutein concentrations. In order to differentiate these response patterns to lutein supplementation, we calculated the rates of change of MPOD levels and serum concentrations of lutein between baseline and six-month supplementation time point for each subject. Whenever the rate of MPOD change was 1.2 or higher (i.e. 20% increase), the MPOD levels were considered “increased” at the six month time point. The value of 1.2 is based on the relative standard deviation of 19.1% for RRS measurements. Whenever the rate of change in serum lutein concentration was 1.1 or higher (i.e. 10% increase), the serum lutein concentration was considered “increased” at the six month time point. The value of 1.1 was adopted from the relative standard deviation of 3.9% with the used measurement technique.

The correlation between baseline MPOD levels and serum lutein concentrations, as well as the rate of MPOD and serum lutein concentration changes, were analyzed with Spearman's rank correlation coefficient test.

## Results

The subject demographic data at baseline are shown in Table 3 (S1 Data). No parameters differed significantly between the FloraGLO and XanMax groups. Based on an interview at each MPOD measurement time point, none of the subjects missed taking lutein supplements throughout the 6-month study, and all were included in the analyses. Compliance was confirmed by checking the number of residual capsules for each subject. No adverse events related to the study supplements were reported during the study period.

**Table 3:** Demographic data at baseline.

Parameter	FloraGLO Group (n = 18)	XanMax Group (n = 18)	*P* Value
Age (years)			
range	20–62	23–62	
Mean ± SD	40.7 ± 13.0	42.2 ± 12.7	0.7191^§^
Gender			
Male/female	9/9	9/9	1.000*
BMI (kg/cm^2^)			
Range	18.8–29.4	19.1–28.6	
Mean ± SD	23.0 ± 3.4	22.1 ± 2.4	0.3516^§^
Smoking^†^			
Never	14 (77.8%)	13 (72.2%)	0.8876^¶^
Past light	2 (11.1)	2 (11.1)	
Past heavy	2 (11.1)	3 (16.7)	
Spherical equivalent refractive error (D)			
Range	-4.9 - +3.0	-5.4 - +0.9	
Mean ± SD	-2.0 ± 1.9	-2.7 ± 2.1	0.2642^§^

SD = standard deviation; BMI = body mass index; D = diopters.

^†^Past light; smoking fewer than 10 daily, Past heavy; smoking 10 or more cigarettes daily

*P* values were calculated to compare the FloraGLOand XanMax groups using either the unpaired *t*-test^§^, Fisher’s exact probability test*, or the G square test^¶^.

### Changes of Mean MPOD Levels in Subjects of FloraGLO and Xanmax Groups

**Fig 2:**
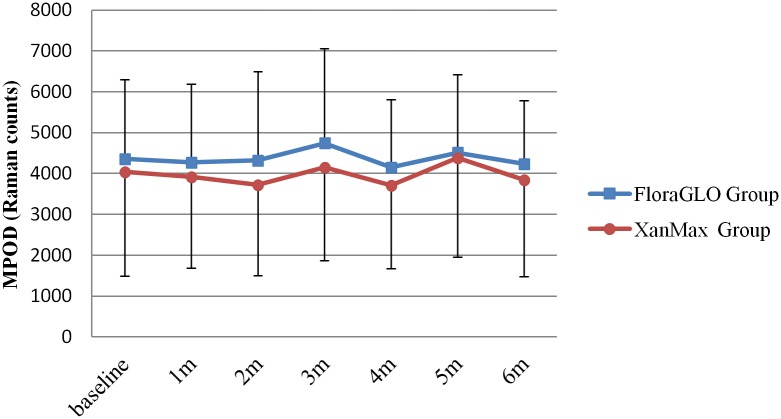
Changes of macular pigment optical density (MPOD) levels in subjects taking two kinds of lutein supplementation. No significant increase was noted in MPOD levels in either group after six months of supplementation.

**Fig 3:**
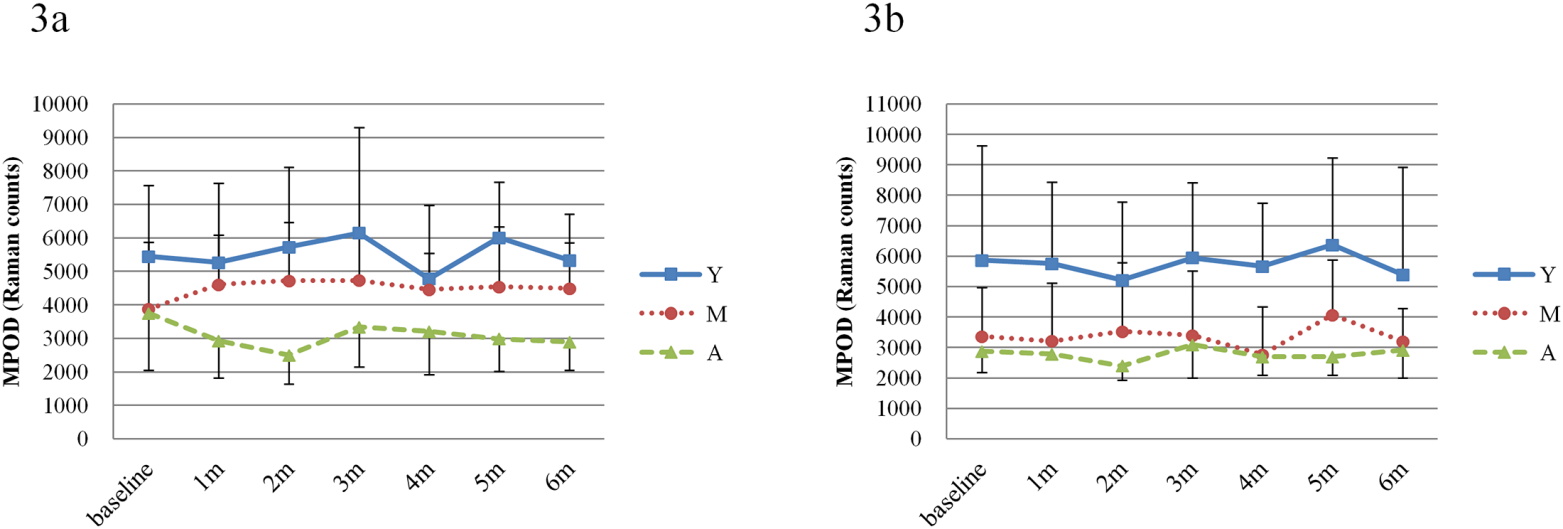
Changes in macular pigment optical density (MPOD) levels for three different age ranges in the FloraGLO group (a) and the XanMax group (b). No significant increase was noted in MPOD levels in either group. The mean MPOD levels in the highest age group were lower than that in the youngest group. (Y; age range from 20 to 34 years old, M; age range from 35 to 49 years old, A; age range of 50 years old and higher).

Mean MPOD levels (in Raman counts) at baseline were 4358±1936 in the FloraGLO group and 4039±2546 in the XanMax group. There was no statistically significant difference between them (*P* = 0.6832, unpaired t-test). The changes of MPOD levels in either group are shown in Fig 2 (S2 Data). After the start of supplementation, the MPOD levels were almost equivalent up to the end of supplementation in either group (*P* = 0.4957 for the FloraGLO group and *P* = 0.4878 for the XanMax group, MANOVA, S1 File). During the supplementation period, the levels of MPOD were not significantly different between FloraGLO and XanMax groups (*P* = 0.9788, MANOVA). Fig 3a and 3b (S3 Data) show the changes of MPOD levels in three groups of different age range, and there were no significant increases in MPOD levels after supplementation in either group. There was a tendency for MPOD levels to decline with increasing age. The mean MPOD levels in the highest age group were lower than those in the youngest group, but this was not statistically significant (*P* = 0.4830 for the FloraGLO group and *P* = 0.0997 for the XanMax group, MANOVA).

### Changes of Serum Lutein Concentration in FloraGLO and XanMax Groups

**Fig 4:**
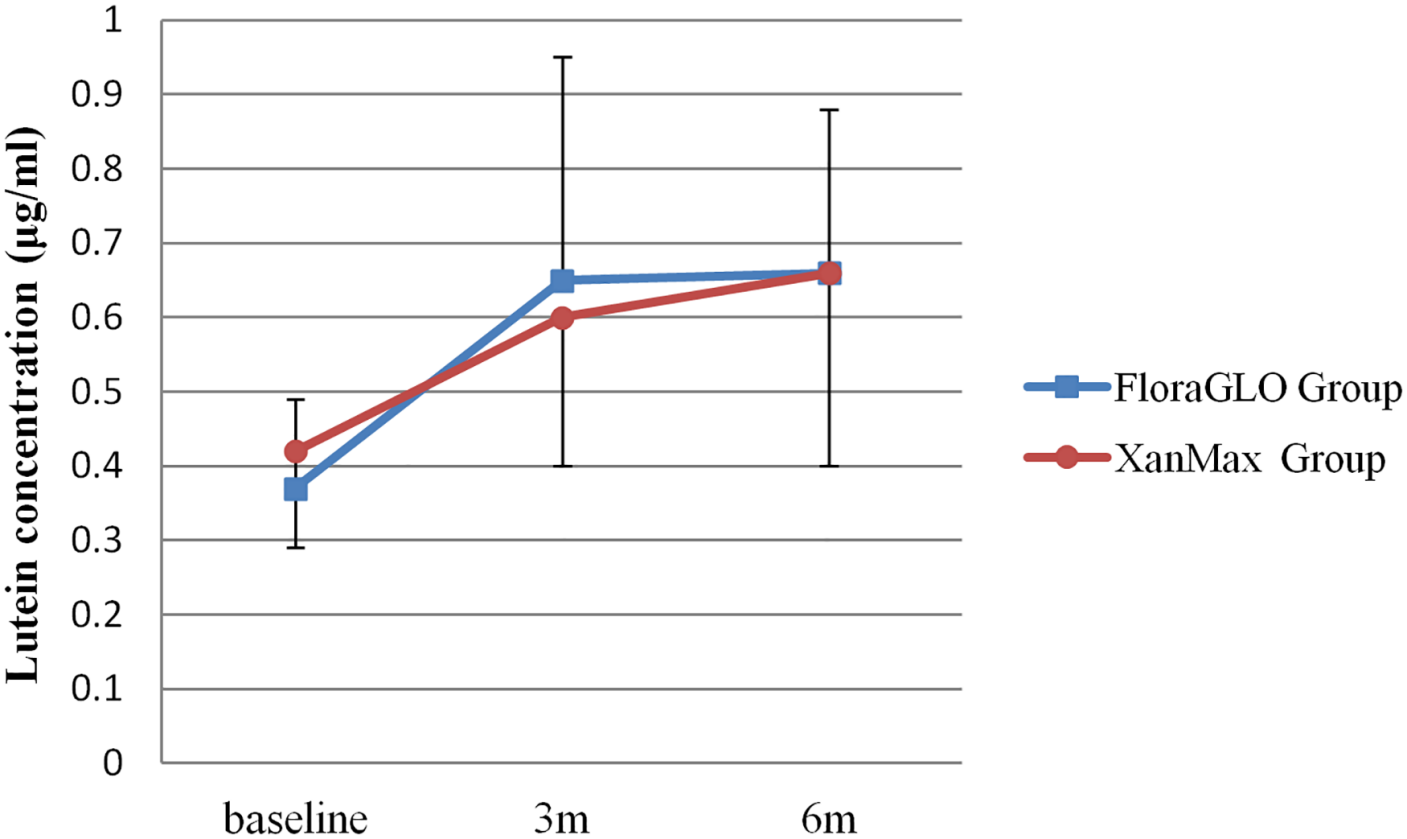
Changes in serum concentration of lutein for subjects taking two kinds of lutein supplementation. Serum concentration significantly increased at three months after supplementation, and the high levels continued until the end of supplementation in both groups.

The serum concentration of lutein at baseline was 0.37±0.12 μg/ml in the FloraGLO group and 0.42±0.13μg/ml in the XanMax group. There was no statistically significant difference between them (*P* = 0.2029, unpaired t-test). At 3 months after the start of supplementation, the serum concentration increased to 0.65±0.30 μg/ml in the FloraGLO group and to 0.60±0.20 μg/ml in the XanMax group, and these high levels continued until the end of supplementation. Changes in lutein serum concentration are shown in Fig 4 (S4 Data). The increase in serum concentration was statistically significant in both groups (*P* = 0.0008 for the FloraGLO group and P = 0.0016 for the XanMax group, MANOVA, S2 File), but there was no difference in serum concentration between two groups (*P* = 0.3603, MANOVA). No remarkable difference was noted in the pattern of increasing serum lutein concentration in the three age groups (data not shown).

There was no significant correlation between MPOD levels and serum lutein concentration in either group at baseline (FloraGLO; rs = -0.23, *P* = 0.3644, XanMax; rs = 0.18, *P* = 0.4651), month 3 (FloraGLO; rs = 0.40, *P* = 0.1023, XanMax; rs = 0.01, *P* = 0.9643), and month 6 (FloraGLO; rs = 0.07, *P* = 0.7976, XanMax; rs = 0.39, *P* = 0.1112).

### Three Response Patterns for the Increase of MPOD Levels and Serum Concentrations of Lutein upon Supplementation

**Fig 5:**
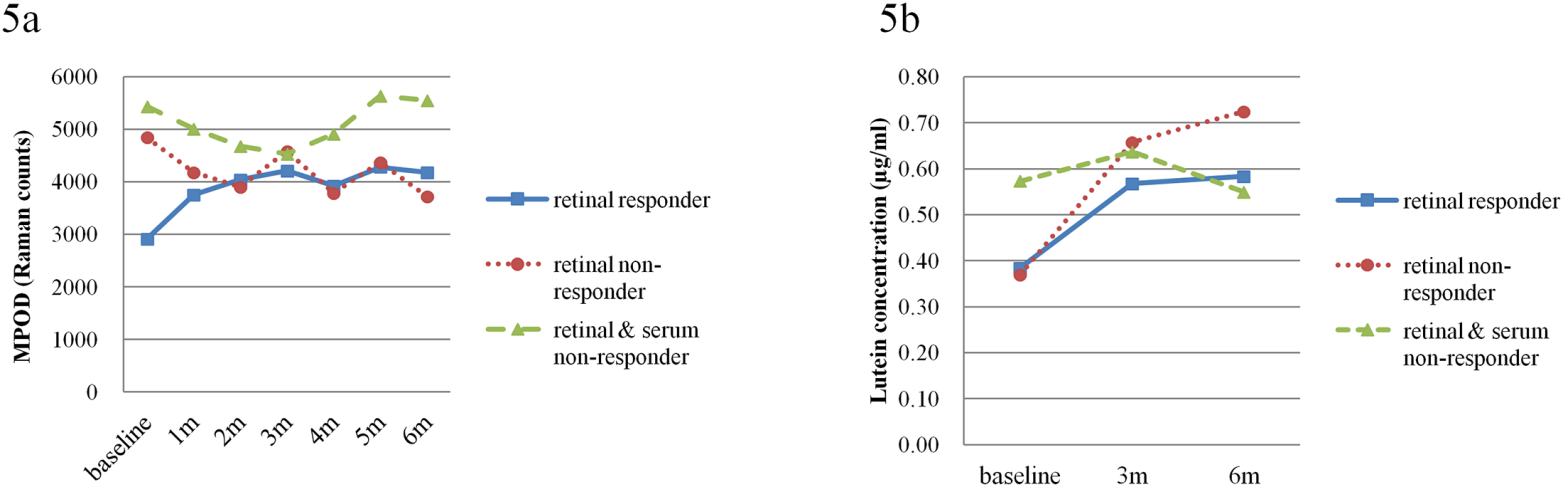
Changes of MPOD levels (a) and serum lutein concentration (b) in three different response pattern groups. The subjects represented by the solid line showed increases in both MPOD levels and serum lutein concentrations. These subjects were designated “retinal responders”. The subjects represented by the dotted line had no increase in MPOD levels but had increases in serum lutein concentrations. These subjects were designated “retinal non-responders”. The subjects represented by the broken line had no increase in both MPOD levels and serum lutein concentrations. These subjects were designated “retinal and serum non-responders”.

Although the mean MPOD levels showed no increase with supplementation, some individuals did in fact show an increase in MPOD levels, and the responses of MPOD levels and serum lutein concentrations to supplementation could be divided into three patterns. Thirteen subjects (FloraGLO group 8, XanMax group 5) showed increases in both MPOD levels and serum concentrations of lutein. These 13 subjects appeared to correspond to “retinal responders” according to the classification of Hammond et al [15]. Twenty subjects (FloraGLO group 9, XanMax group 11) showed an increase in serum concentration but not in MPOD level, corresponding to “retinal non-responders”. Three subjects (FloraGLO group 1, XanMax group 2) did not show any increases in either MPOD levels or plasma concentrations, and correspondingly were classified as “retinal and serum non-responders”. The changes in MPOD levels and serum concentrations of the three response patterns are shown in Fig 5 (S5 Data).

Table 4 shows further characteristics of the three response patterns. Since the number of retinal and serum non-responders was small, the statistical comparisons were performed between retinal responders and retinal non-responders only. There were no significant differences in age, kind of lutein supplement, BMI, smoking habits, or refractive errors between them. However, the MPOD levels at baseline in retinal non-responders were significantly higher than in retinal responders (2919 ± 993 in retinal responders, 4845 ± 2390 in retinal non-responders, *P* = 0.0034, unpaired t-test). There was a significant difference in gender (*P* = 0.0377, Fisher’s exact probability test). Men were predominant in retinal-responders.

**Table 4:** Demographic Data of the Three Response Groups.

Parameter	Retinal Responder (n = 13)	Retinal Non-responder (n = 20)	*P* Value	Retinal and serum non-responder (n = 3)
Age (years)				
Mean ± SD	42.2 ± 12.6	40.6 ± 12.7	0.7247^§^	44.3
Gender				
Male/female	9/4	6/14	0.0377*	3/0
Lutein supplement				
FloraGLO/XanMax	8/5	9/11	0.4813*	1/2
BMI (kg/cm^2^)				
Mean ± SD	22.5 ± 3.0	22.5 ± 3.2	0.9816^§^	23.0
Smoking^†^				
Never	8 (61.5%)	18(90%)	0.1468^¶^	1
Past light	2 (15.4)	1 (5.0)		1
Past heavy	3 (23.1)	1 (5.0)		1
Spherical equivalent refractive error (D)				
Mean ± SD	-2.9 ± 2.1	-2.1 ± 2.0	0.2993^§^	-1.7
MPOD levels at baseline				
Mean ± SD	2919 ± 993	4845 ± 2390	0.0034^§^	5431
Serum lutein concentration at baseline				
Mean ± SD	0.39 ± 0.12	0.37 ± 0.12	0.7308^§^	0.57

SD = standard deviation; BMI = body mass index; D = diopters.

^†^Past light; smoking fewer than 10 daily, Past heavy; smoking 10 or more cigarettes daily

*P* values were calculated to compare the FloraGLOand XanMax groups using either the unpaired *t*-test^§^, Fisher’s exact probability test*, or the G square test^¶^.

There was no difference in serum lutein concentration between retinal responders and retinal non-responders, but the serum lutein concentrations in retinal and serum non-responders were higher than those in retinal responders and non-responders.

### Correlation between baseline values of MPOD and serum lutein concentrations and changes in MPOD and serum lutein concentration

Fig 5 shows a tendency for MPOD levels to increase in subjects with low baseline MPOD levels, and similarly a tendency for serum lutein concentrations to increase in subjects with low lutein baseline concentrations. Therefore, we analyzed the correlations between baseline MPOD levels and serum lutein concentrations and the rate of MPOD levels and serum lutein concentration changes (Tables 5 and 6). Baseline MPOD levels negatively correlated with the changes in MPOD level during 3 and 6 months, i.e., the subjects with low MPOD levels at baseline tended to increase MPOD levels during 3 and 6 months. Serum lutein concentration at baseline negatively correlated with the changes in lutein concentration during 6 months, i.e., the subjects with low serum lutein concentration at baseline tended to increase serum lutein concentration during 6 months.

**Table 5:** Correlation between baseline values of MPOD and serum lutein concentrations and changes in MPOD and serum lutein concentration at 3 months.

	MPOD at baseline	MPOD change at 3 months	Serum lutein concentration at baseline	Serum lutein concentration change at 3 months
MPOD at baseline	-	*p* = 0.0268*	*p* = 0.8744	*p* = 0.3222
Rate of MPOD change at 3 months	r = -0.3189*	-	*p* = 0.4238	*p* = 0.5708
Serum lutein concentration at baseline	r = -0.0273	r = -0.1375	-	*p* = 0.1376
Rate of serum lutein concentration change at 3 months	r = 0.1698	r = 0.0977	r = -0.2523	-

Rate of MPOD change at 3 months = (MPOD at 3 months—MPOD at baseline)/MPOD at baseline. Rate of serum lutein concentration change at 3 months = (serum lutein concentration at 3 months—serum lutein concentration at baseline)/serum lutein concentration at baseline. The correlation coefficient (r) and *p* values are calculated by Spearman's rank correlation coefficient. The * indicates *p*<0.05.

**Table 6:** Correlation between baseline values of MPOD and serum lutein concentrations and changes in MPOD and serum lutein concentration at 6 months.

	MPOD at baseline	MPOD change at 6 months	Serum lutein concentration at baseline	Serum lutein concentration change at 6 months
MPOD at baseline	-	*p* = 0.0133*	*p* = 0.8744	*p* = 0.0852
Rate of MPOD change at 6 months	r = -0.4090*	-	*p* = 0.762	*p* = 0.1945
Serum lutein concentration at baseline	r = -0.0273	r = 0.0523	-	*p* = 0.0001**
Rate of serum lutein concentration change at 6 months	r = 0.2909	r = -0.2214	r = -0.5919**	-

Rate of MPOD change at 6 months = (MPOD at 6 months—MPOD at baseline)/MPOD at baseline. Rate of serum lutein concentration change at 6 months = (serum lutein concentration at 6 months—serum lutein concentration at baseline)/serum lutein concentration at baseline. The correlation coefficient (r) and *p* values are calculated by Spearman's rank correlation coefficient. The * and ** indicate *p*<0.05 and *p*<0.01, respectively.

### Functional Assessment

**Fig 6:**
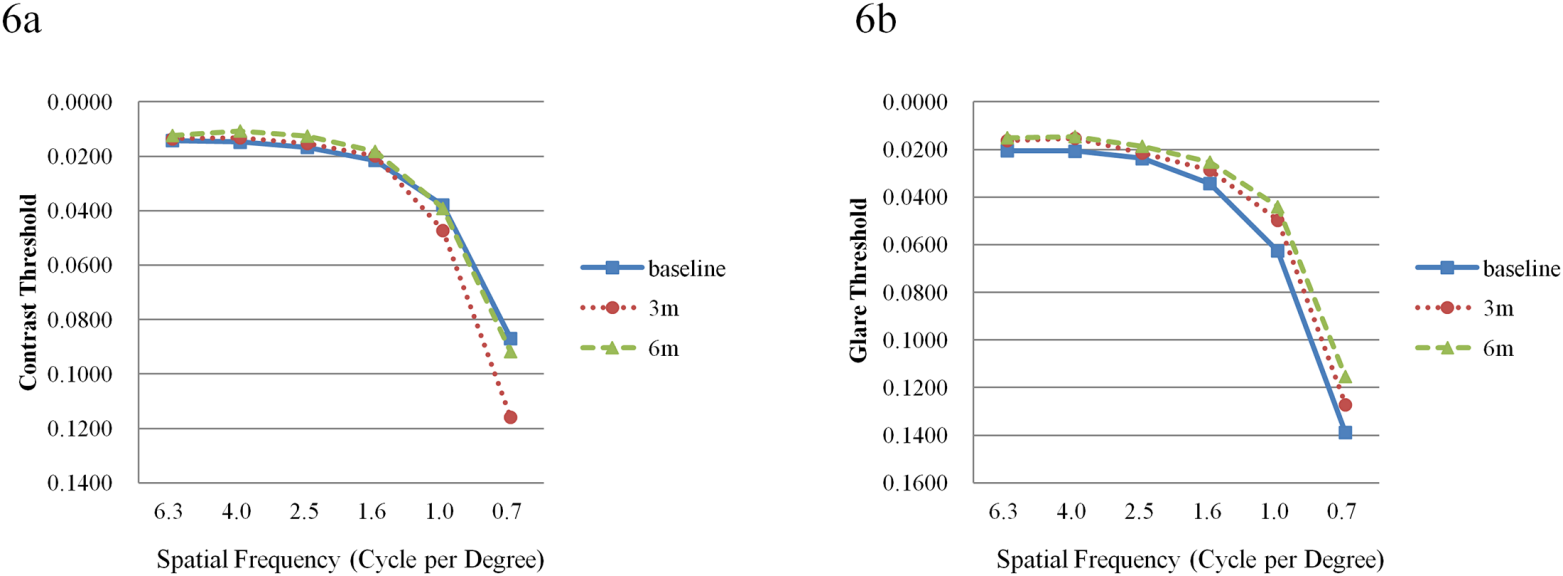
Contrast (a) and glare (b) threshold values in retinal responders. a. The transverse axis represents the size (visual angle) of the target. No statistically significant improvements were noted across all targets except for 6.3 degrees between baseline and 6 months later. b. Glare sensitivities were significantly improved at the target size of 4.0, 2.5, 1.6 and 1.0 degree between baseline and six months later.

Contrast and glare sensitivities in retinal responders are shown in Fig 6a and 6b (S6 Data). At month 6, no statistically significant improvements were noted in contrast sensitivity across all targets except for 6.3 degrees (*P* = 0.0010), the largest target size of the instrument. At month 6, glare sensitivities were significantly improved at the target size of 4.0, 2.5, 1.6 and 1.0 degree (*P* = 0.0429, 0.0358, 0.0437, 0.0137) and marginally improved at 6.3 degree (*P* = 0.0535). In retinal non-responders, contrast and glare sensitivity showed no significant improvements at six months at all size of targets with one each exception at 6.3 degree (*P* = 0.0010) in contrast sensitivity and 4.0 (*P* = 0.0207) in glare sensitivity.

Macular ERG results are shown in Table 7 (S7 Data). There were no significant differences of a-wave and b-wave latency and amplitude between baseline and 6 months afterwards in retinal responders and retinal non-responders (paired t-test).

**Table 7:** Latency and Amplitude of Macular ERG.

	Retinal responder			Retinal non-responder		
	baseline	6 months	*P* Value	baseline	6 months	*P* Value
Latency of a-wave						
Mean ± SD	26.6 ± 1.1	26.9 ± 1.1	0.4880	26.1 ± 1.4	26.3 ± 1.1	0.4101
Amplitude of a-wave						
Mean ± SD	-1.1 ± 0.4	-1.3 ± 0.6	0.1891	-1.4 ± 0.4	-1.2 ± 0.9	0.3184
Latency of b-wave						
Mean ± SD	45.9 ± 3.1	46.4 ± 3.0	0.6416	46.2 ± 2.5	46.5 ± 2.3	0.7443
Amplitude of b-wave						
Mean ± SD	3.0 ± 1.1	3.2 ± 0.8	0.1944	3.7 ± 0.9	3.6 ± 0.6	0.6792

SD = standard deviation

## Discussion

The changes in MPOD levels and serum lutein concentrations with two kinds of supplementation containing same amounts of lutein but with different particle sizes and slightly different amounts of zeaxanthin have been investigated in this Asian population. The mean serum lutein concentrations increased, but the mean MPOD levels showed no increase in either supplement group. The mean MPOD levels also did not change when the three age groups were analyzed, although there was a tendency that MPOD levels in the older group were lower than in the younger group. No significant correlation between serum lutein concentration and MPOD levels were observed, as shown in previous studies [16,17]. Thus, FloraGLO and XanMax appear to increase serum lutein concentration equally and to have a similar effect on MPOD levels. We consider it likely that the difference in zeaxanthin content of 0.33 mg (Flora GLO;0.96 mg, XanMax; 1.29 mg) is too small to have a differential effect on increasing MPOD levels in six months. The lutein particle size of FloraGLO is larger than that of XanMax. Generally, smaller sized lutein particles are thought to be more easily absorbed in the intestine; however, the similar effect of these two products suggests that the particle size of FloraGLO is sufficiently small for efficient absorption.

Although the mean MPOD levels in the present subjects did not change, a more detailed analysis showed that thirteen subjects (FloraGLO group 8, Xanmax group 5) showed increases in MPOD levels at one month after supplementation and continued gradual increases up to the end of supplementation (Fig 5). The serum lutein concentration of these subjects also increased. Depending on the change in MPOD levels and serum lutein concentration, the subjects could be divided into three groups, as suggested by Hammond et al [15]. They examined MPOD levels and serum concentrations of lutein in 13 subjects who had been fed a lutein and zeaxanthin rich diet. They identified eight “retinal responders”, who had increases in serum lutein and MPOD levels, two “retinal non-responders”, who had increases in serum lutein but not in MPOD levels, and one “retinal and serum non-responder”, who had no increase in either serum lutein or MPOD level. Following this classification, our results contained 13 “retinal responders”, 20 “retinal non-responders”, and 3 “retinal and serum non-responders”. There were no significant differences in demographic characteristics among the three groups (Table 3); however, the MPOD levels at baseline in the “retinal responders” were significantly lower than MPOD levels in “retinal non-responders” (*P* = 0.001, t-test, Fig 5a, Table 4). The serum lutein concentration at baseline did not differ between them, and serum lutein concentrations of both groups were lower than those in “retinal and serum non-responders” (Fig 5b). These results indicate that retinal responders have relatively low serum lutein concentrations and low MPOD levels at baseline, and that serum lutein concentrations and MPOD levels increased with lutein supplementation. Retinal non-responders have low serum lutein concentration and relatively high level of MPOD which might mean that they have already reached a saturation point before supplementation, as suggested by Connolly et al [18]. The fact that MPOD levels in retinal responders were lower than in non-responders, despite having equivalent serum lutein concentrations, suggests the possibility of poor uptake of lutein into the retina by retinal responders. In other words, retinal responders and non-responders might absorb lutein equally in the intestine, but the mechanisms of uptake and accumulation of lutein in the retina may be relatively insufficient in retinal responders compared to retinal non-responders. In 18 of 23 retinal non-responders, MPOD levels at six months after the start of supplementation was slightly lower than at baseline. This negative response to supplementation was also noted by Hammond et al [15], who suggested the possibility that persons could respond negatively to dietary modification. The true reason of this negative response remains unknown, and further study might be needed.

The number of retinal and serum non-responders was too small for a statistical analysis, but these subjects had higher serum lutein concentrations and MPOD levels at baseline compared to the other two groups (Fig 5b). This fact suggests that retinal and serum non-responders might already have taken up enough lutein via daily diet, allowing them to reach a saturation point of MPOD levels, assuming serum lutein concentration reflects dietary lutein consumption.

Considering the obtained results, the terms “retinal responder”, “retinal non-responder” and “retinal and serum non-responder” may not be appropriate to describe the character of the three response patterns. Retinal responders are subjects whose dietary consumption of lutein is insufficient to maintain high levels of MPOD. Therefore, they might be good candidates for lutein supplementation. Retinal non-responders are subjects who have a high ability for carotenoid uptake into the retina even with relatively small consumption of dietary lutein. Retinal and serum non-responders are subjects who have already high MPOD levels from sufficient dietary lutein consumption. These conclusions are supported by Tables 5 and 6, showing that baseline MPOD levels and serum lutein concentrations had a negative correlation with MPOD levels and lutein concentrations at 3 or 6 months. Subjects with low MPOD levels at baseline and subjects with low lutein concentration were expected to increase MPOD levels and lutein concentrations by taking supplements, respectively.

The mean rate of MPOD level increase for retinal responders was 46% and 26% in the FloraGLO and XanMax groups, respectively. Our previous study measuring MPOD levels with the same instrument after 10 mg of FloraGLO lutein supplementation for three months showed a rate of MPOD level increase 〔 = (MPOD at the end of study − MPOD at baseline) / MPOD at baseline ×100%)〕of 24% for the average of 11 subjects [19]. The difference between 47% in the present study and 24% in the previous study might occur because subjects in the previous study contained some retinal non-responders. The mean rates for MPOD level increase for retinal responders in Caucasians have been reported to be 16–28% in healthy subjects, and 12–51% in AMD patients, although the supplement formulations and MPOD measurement methods were not identical [15,17,20–24]. The present results with FloraGLO and XanMax and RRS measurement were comparable to these reports.

Functional changes were assessed with contrast and glare sensitivity tests and macular ERGs. The relationship between macular pigment and visual functions have been previously investigated, with several reports suggesting an improvement of contrast sensitivity [6,22], glare sensitivity [25], and other functions such as photostress recovery and visual discomfort [5,6,7,26], although some studies have failed to show any effectiveness [27]. In the present study, there were no remarkable changes in contrast sensitivity, while glare sensitivity did improve in retinal responders. The glare sensitivity measured by CGT-1000 is considered a type of disability glare, and this improvement was consistent with the results of Stringham et al [25]. Falsini [28] demonstrated that lutein and other antioxidant supplementation led to improvements in the amplitude of focal ERGs in 17 early age-related maculopathy patients. The functional improvement in the central retina was also observed by multifocal ERG [29], and Ma et al [6] suggested a significant association between the change in MPOD and the change in N1P1 response densities. However, in the present study, there were no changes in the latency or amplitude of macular ERGs in subjects with increased MPOD levels after supplementation. The present subjects had normal eyes with good contrast and glare sensitivities and macular ERG. This high sensitivity and good response in macular ERG might be one reason for the difficulty to show improvements of contrast and glare sensitivities and macular ERG results.

A shortcoming of the present study is a lack of information on genotype and dietary intakes of carotenoids for each subject. This is important because not only diet can influence ocular carotenoid status but there is also a well-known hereditability of macular pigment levels [30]. Genetic variants related to carotenoid metabolism have recently been reported to modulate macular pigment levels [31,32]. The present study was not designed originally as an equivalent trial for two lutein supplements. Therefore, the equality of FloraGLO® and Xanmax® on MPOD levels and serum lutein concentrations could not be proven. However, based on the results obtained from this study, we can state that there was no statistically significant difference of MPOD levels and serum lutein concentrations between these two lutein supplements in the measured subjects. The responses of MPOD levels and serum lutein concentrations to supplementation could be reasonably divided into three patterns, and subjects with relatively low MPOD levels at baseline tended to show an increase of MPOD levels upon lutein supplementation. This is an important message for physicians recommending supplementation to their patients. It may be appropriate to consider the particular MPOD level and serum lutein concentration for each patient when recommending lutein supplements. However, since the number of subjects was low, a further study with more subjects is needed to prove that subjects with low MPOD levels will benefit from lutein supplementation.

## Supporting Information

S1 CONSORT Checklist CONSORT Checklist. (DOCX)

S1 Data Data underlying the findings. (XLSX)

S2 Data Data for Fig 2. (XLSX)

S3 Data Data for Fig 3. (XLSX)

S4 Data Data for Fig 4. (XLSX)

S5 Data Data for Fig 5. (XLSX)

S6 Data Data for Fig 6. (XLSX)

S7 Data Data for Table 7. (XLSX)

S1 File MPOD. (DOCX)

S2 File Serum lutein conc. (DOCX)

S1 Protocol Study protocol (original). (DOCX)

S2 Protocol Study protocol (English). (DOCX)
