# Supplementary material for: Changes in Macular Pigment Optical Density and Serum Lutein Concentration in Japanese Subjects Taking Two Different Lutein Supplements
Source: PLoS One. 2015 Oct 9;10(10):e0139257. doi: 10.1371/journal.pone.0139257 (PMC4599964; doi:10.1371/journal.pone.0139257)
Supplement: S1 File — (DOCX) [file pone.0139257.s009.docx]

S8. Script for comparison in MPOD levels between two supplemental groups.

Fit Model**(**

Y**(**

:Name**(** "0baseline" **)**,

:Name**(** "1m" **)**,

:Name**(** "2m" **)**,

:Name**(** "3m" **)**,

:Name**(** "4m" **)**,

:Name**(** "5m" **)**,

:Name**(** "6m" **)**

**)**,

Effects**(** :group **)**,

Personality**(** Manova **)**,

Run**(**

Response Function**(**

Sum,

Test Each Column Separately Also,

Repeated**(** **1** **)**,

Title**(** "Between Subjects" **)**

**)**,

Response Function**(**

Contrast,

Univariate Tests Also,

Test Each Column Separately Also,

Repeated**(** **2** **)**,

Prefix**(** "period*" **)**,

Title**(** "Within Subjects" **)**

**)**

**)**

**)**;
