# Supplementary material for: Changes in Macular Pigment Optical Density and Serum Lutein Concentration in Japanese Subjects Taking Two Different Lutein Supplements
Source: PLoS One. 2015 Oct 9;10(10):e0139257. doi: 10.1371/journal.pone.0139257 (PMC4599964; doi:10.1371/journal.pone.0139257)
Supplement: S1 Protocol — (DOCX) [file pone.0139257.s011.docx]

2010/05/23作成

2010/7/12修正

**臨床研究計画書**

研究課題名：**2種類のルテインサプリメントの黄斑色素と視機能に対する影響**

臨床研究依頼者

聖隷浜松病院眼科

代表責任者　　　　　　尾 花　明

# **1．研究の概要と意義**

　網膜中心部である黄斑には2種類のカロチノイドすなわちルテインとゼアキサンチンからなる黄斑色素が存在する。ルテインとゼアキサンチンは図1の構造をもち、両者は二重結合の位置が一箇所異なる。ゼアキサンチンには3つの立体異性体があるが、網膜にはその内の(3R,3’R)ゼアキサンチンと(3R,3’S) ゼアキサンチン（メソゼアキサンチン）が存在する。


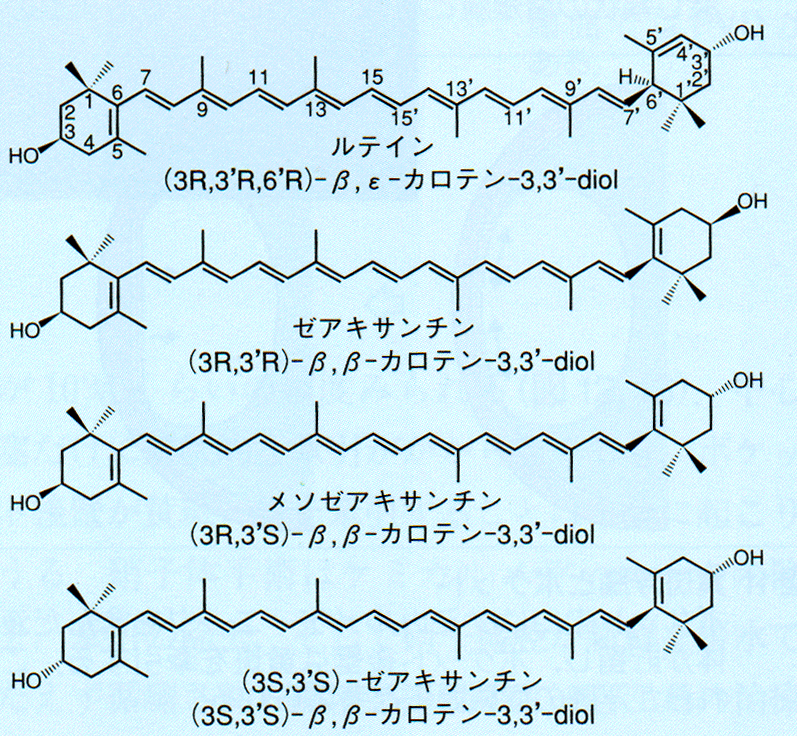
　図１　ルテインとゼアキサンチンの分子構造

　食事で摂取されたルテインと(3R,3’R)ゼアキサンチンは小腸上皮で吸収されて肝臓でリポ蛋白に組み込まれて血液中にでる。血漿中のルテイン、ゼアキサンチンは脈絡膜毛細血管から網膜色素上皮を介して視細胞外節に取り込まれて軸索に集まり、内網状層にもたまる。ゼアキサンチンの結合タンパクはイソ型のglutathione S-transferaseP1(GSTP1)であり、GSTP1は錐体軸索に分布するため錐体軸索である黄斑の外網状層（ヘンレ線維層）にゼアキサンチンは多く存在する。なお、ルテインはGSTP1には結合せず、結合タンパクは23kDa付近のタンパクとされる。ルテインは黄斑周囲の杆体分布領域に多いが、軸索以外に外節にも確認されている。なお、(3R,3’S) ゼアキサンチンは食品中には含まれず、網膜内でルテインから変換される。

　近年、黄斑色素が臨床的に注目される理由として加齢性黄斑変性との関係が上げられる。加齢性黄斑変性は欧米諸国では以前から高齢者の失明疾患として重要視されていたが、近年はわが国を含むアジア諸国でも増加している。加齢性黄斑変性は萎縮型と滲出型に分けられるが、前者には有効な予防法と治療法がなく、後者には近年光線力学療法と抗血管内皮増殖因子阻害剤の硝子体内注射治療が盛んに行われるものの、あくまでも病気の進行を抑えるのみで根治はできず、しかも年余にわたって治療を継続する必要がある。加齢性黄斑変性では光も失うような完全失明に至る症例はまれではあるが、患者は視野中央の影（暗点）やゆがみ（変視症）と視力低下のために生活の質の著しい低下に苦しむ。加齢性黄斑変性は加齢、遺伝形質、環境要因、生活習慣など複数の要因によって発症するが、根本的要因として以下に記載した光による酸化ストレスがあげられる。

　ヒトの眼は視細胞外節円板の膜タンパクである視色素が光を受けて構造変化を起こすことで生じた膜電位差を視覚情報として脳に送る。このとき変化した視色素を含む外節円板は膜の恒常性を失い細胞体から脱落して網膜色素上皮に貪食される。貪食された円板はライソゾーム酵素で消化されるが、消化しきれずに残った残余小体は加齢とともに増加し、網膜色素上皮細胞内に蓄積してリポフスチン顆粒となる。リポフスチンはブルッフ膜にも蓄積する。リポフスチンはラジカル発生源となり、ヒトがものを見るという行為を行う限り、必ず光を吸収したリポフスチンから活性酸素が発生し酸化ストレスにさらされる。また、視細胞ミトコンドリアのチトクロームCやフラビン、レチナールも光励起によりラジカルを発生することが示唆されている。これらの活性酸素は視細胞や網膜色素上皮細胞の細胞膜を障害する。網膜色素上皮細胞内の抗酸化物質と視細胞外節に存在するルテインはこの活性酸素を除去して視細胞障害を抑制することが複数の実験で実証されている。また、網膜内外網状層にあるルテインとゼアキサンチンは青色光を吸収し、フィルター効果によってリポフスチンに達する青色光を減少させて活性酸素の発生自体を抑制する。このように黄斑色素は網膜視細胞の恒常性維持に重要な役割を果たすが、生体にとって不都合なことにリポフスチンは加齢とともに増加し、黄斑色素は加齢とともに減少し、酸化と抗酸化のバランスが崩れたときに加齢黄斑変性を代表とする眼疾患が発症する。

　黄斑色素には防御機能以外に視機能にも影響する可能性がある。たとえば、健常高齢者で黄斑色素密度が高いと網膜感度が高いという報告や、加齢黄斑症患者がルテイン摂取で黄斑色素が増加すると多局所網膜電位図の振幅が増大したとの報告がある。早期加齢黄斑症患者ではルテイン摂取でコントラスト感度の一部が改善したとの報告もあるが、一般的にその効果は確定していない。

　我々はこれまでにヒトを対象に行った臨床研究にて、健常者の黄斑色素密度は加齢とともに減少すること、男性は女性よりも少ないこと、加齢性黄斑変性患者の患眼は健常者より少ないこと、さらに患者の僚眼で黄斑変性に至っていない眼の色素密度も少ないことを報告してきた。また、健常者がルテインサプリメント（10mg/日）を摂取すると血漿ルテイン濃度と黄斑色素密度が上昇すること、しかし、加齢黄斑変性患者では有意な上昇が見られないという結果を得た。一方、健常者がゼアキサンチン(10mg/日)を摂取しても黄斑色素密度は上昇しなかった。これらの試験で使用したルテインはフリー体ルテインである米国ケミン社製のフローラ GLOであった。フリー体ルテインの吸収は、まず、消化管内でルテインが脂肪内に分散し、脂肪の消化が進行してミセルが形成されるとルテインはミセル内に存在した状態で小腸上皮から吸収される。したがって、フリー体ルテインの吸収には脂肪に対する分散のしやすさやミセル内への取り込まれやすさに影響される。脂肪への取り込みには粒子の大きさが関与し、粒子径が小さいほど吸収率がよいと推測される。実際、ラットによる吸収実験では、フローラ GLOより粒子径の小さなザンマックス2002（カトラフィトケム社製）の方が吸収のよいことが示されている。

　そこで、本試験ではすでにその効果が実証されているフローラ GLOとザンマックス2002の効果を比較することを主たる目標とし、また、黄斑色素増加による視機能への影響を副次的評価目標とした。

# **2．研究の目的**

　異なるルテイン原末であるザンマックス2002とフローラ GLOからなる２種類のルテインサプリメントが、健常人の黄斑色素密度と血漿濃度増加効果において同等性を有するか否かを検証する。また、健常眼で黄斑色素が増加することにより網膜機能向上が得られるか否かを探索する。

# **3．対象**

## 3.1．対象者

日本人健常篤志者

聖隷浜松病院職員およびその関係者

以下の選択基準を満たす眼を有すること。

## 3.2．選択基準

重篤な眼疾患を有さない

矯正視力が0.8以上である

屈折度数が等価球面度数-6.0D未満である

消化吸収不良症候群や胃切除などの吸収障害につながる消化器疾患に罹患していない

糖尿病に罹患していない

ルテイン、ゼアキサンチンサプリメントを常用していない

非喫煙者または過去1年以内に喫煙歴がない

本試験の趣旨を理解し、文書による同意が得られる

スクリーニング検査の結果、担当医師が対象として適格と判断できる

ルテインおよびゼアキサンチンアレルギーがない

## 3.3．除外基準

測定に支障を来たす程度の白内障を有する眼

散瞳時の瞳孔径6.5mm未満の散瞳不良眼

その他、試験責任医師が不適当と判断した場合

## 3.4．実施目標症例数

## 表１のごとく合計36名とする。

## 表１　目標症例数

|  | | ザンマックス | | フローラGLO | | 計 |
| --- | --- | --- | --- | --- | --- | --- |
|  |  | 男 | 女 | 男 | 女 |  |
| Y群 | 20-34歳 | 3 | 3 | 3 | 3 | 12 |
| Ｍ群 | 35-49歳 | 3 | 3 | 3 | 3 | 12 |
| Ｓ群 | 50-64歳 | 3 | 3 | 3 | 3 | 12 |
| 計 | | 9 | 9 | 9 | 9 | 36 |

# **4．試験方法**

## 4.1．試験デザイン

　並行群間比較試験

## 4.2．試験手順

試験責任医師または試験分担医師は、試験実施前に被験者に対して問診および眼科的検査（遠見視力測定、眼圧測定、散瞳下での細隙灯顕微鏡検査・眼底検査）を行い、対象眼の選択と選択基準・除外基準に合致するか否かを判断し、試験責任医師が対象眼の合否を決定する。

試験該当者には試験内容の十分な説明を行い、文書による同意を取得する。

同意取得後に被験者登録を行う。このときに被験者番号を決定するが、番号は同意取得順とする。

試験責任医師は以下の表によって被験者番号ごとの被験者の氏名、ID番号を管理する。

|  | 氏名 | ID | 同意取得日 | 試験開始日 |  | 氏名 | ID | 同意取得日 | 試験開始日 |
| --- | --- | --- | --- | --- | --- | --- | --- | --- | --- |
| Y-male1 |  |  |  |  | Y-female1 |  |  |  |  |
| Y-male2 |  |  |  |  | Y-female2 |  |  |  |  |
| Y-male3 |  |  |  |  | Y-female3 |  |  |  |  |
| Y-male4 |  |  |  |  | Y-female4 |  |  |  |  |
| Y-male5 |  |  |  |  | Y-female5 |  |  |  |  |
| Y-male6 |  |  |  |  | Y-female6 |  |  |  |  |
|  | | | | | | | | | |
| M-male1 |  |  |  |  | M-female1 |  |  |  |  |
| M-male2 |  |  |  |  | M-female2 |  |  |  |  |
| M-male3 |  |  |  |  | M-female3 |  |  |  |  |
| M-male4 |  |  |  |  | M-female4 |  |  |  |  |
| M-male5 |  |  |  |  | M-female5 |  |  |  |  |
| M-male6 |  |  |  |  | M-female6 |  |  |  |  |
|  | | | | | | | | | |
| S-male1 |  |  |  |  | S-female1 |  |  |  |  |
| S-male2 |  |  |  |  | S-female2 |  |  |  |  |
| S-male3 |  |  |  |  | S-female3 |  |  |  |  |
| S-male4 |  |  |  |  | S-female4 |  |  |  |  |
| S-male5 |  |  |  |  | S-female5 |  |  |  |  |
| S-male6 |  |  |  |  | S-female6 |  |  |  |  |

## 4.3．眼科的必須検査

　遠見視力測定、近見視力測定、コントラスト感度測定、グレア感度測定、トライイリス測定、黄斑局所網膜電位図（黄斑ERG）、光干渉断層計(OCT)検査、眼底カラー写真撮影、黄斑色素密度測定

黄斑色素密度測定方法；共鳴ラマン分光装置と眼底自家蛍光分光装置の両方を使用する。被験眼を散瞳剤（サンドール®, 日点またはミドリンP, 参天製薬）にて散瞳する。瞳孔径は7.0mm以上が望ましいが、最低でも6.5mm以上とする。

《共鳴ラマン分光装置》被験者は顎をMacular Pigment Raman Detectorの顎台にのせて頭部を固定する。検者は被験者の眼底を確認しながら測定を行う。測定は計３回行い、すべての値をカルテ上に記録する。

《眼底自家蛍光分光装置》被験者は顎を自家蛍光型黄斑色素測定装置の顎台にのせて青色光の中央部を固視する。験者は被験眼が固視した時点で装置のシャッターを押す。

試験責任医師は記録シートに被検者番号と眼科的検査結果を記載し保管する。

## 4.4．血漿ルテイン濃度の測定

　へパリン添加真空採血管を用い、静脈血約2 mlを採取する。検体は速やかに遠心分離(4℃、3000回転、15分)し、得られた血漿を2本のサンプルチューブに均等に分取する。血漿サンプルは、分析実施施設に送付するまで-20℃以下にて凍結保管する。

測定は以下で行う。

大塚製薬株式会社　診断事業部

〒108-8242東京都港区港南2-16-4品川グランドセントラルタワー12F

電話：03-6361-7304　 FAX: 03-6717-1467

測定結果は同事業部から試験責任医師に送付され、試験責任医師がこれを保管する。

4.5．検査施行スケジュール

表3に従って行う。

表3　検査スケジュール

|  | 開始前 | 1ヵ月目 | 2ヵ月目 | 3ヵ月目 | 4ヶ月目 | 5ヶ月目 | 6ヶ月目 |
| --- | --- | --- | --- | --- | --- | --- | --- |
| 遠・近視力 | ○ |  |  | ○ |  |  | ○ |
| ｺﾝﾄﾗｽﾄ・ｸﾞﾚｱ | ○ |  |  | ○ |  |  | ○ |
| ﾄﾗｲｲﾘｽ | ○ |  |  | ○ |  |  | ○ |
| 黄斑ERG | ○ |  |  |  |  |  | ○ |
| OCT | ○ |  |  |  |  |  | ○ |
| ｶﾗｰ撮影 | ○ |  |  |  |  |  |  |
| 黄斑色素測定 | ○ | ○ | ○ | ○ | ○ | ○ | ○ |
| 採血 | ○ |  |  | ○ |  |  | ○ |

4.6．サプリメンの割り付け方法

割り付け責任者はあらかじめ、各被験者番号にザンマックスかフローラGLOのいずれかを割り付けた表を作製する（表4）。その際、各群の男女で両者の例数が同一になるようにする。たとえばY群男性ではザンマックス3例、フローラGLO3例とする。

表4　割付例（実際のものとは異なる）

|  | 試験食品 |  | 試験食品 |
| --- | --- | --- | --- |
| Y-male1 | ザンマックス | Y-female1 | フローラGLO |
| Y-male2 | ザンマックス | Y-female2 | ザンマックス |
| Y-male3 | フローラGLO | Y-female3 | フローラGLO |
| Y-male4 | フローラGLO | Y-female4 | ザンマックス |
| Y-male5 | ザンマックス | Y-female5 | ザンマックス |
| Y-male6 | フローラGLO | Y-female6 | フローラGLO |
|  |  |  |  |
| M-male1 | ザンマックス | M-female1 | フローラGLO |
| M-male2 | フローラGLO | M-female2 | ザンマックス |
| M-male3 | ザンマックス | M-female3 | ザンマックス |
| M-male4 | フローラGLO | M-female4 | フローラGLO |
| M-male5 | フローラGLO | M-female5 | フローラGLO |
| M-male6 | ザンマックス | M-female6 | ザンマックス |
|  |  |  |  |
| S-male1 | ザンマックス | S-female1 | フローラGLO |
| S-male2 | ザンマックス | S-female2 | ザンマックス |
| S-male3 | ザンマックス | S-female3 | フローラGLO |
| S-male4 | フローラGLO | S-female4 | ザンマックス |
| S-male5 | フローラGLO | S-female5 | フローラGLO |
| S-male6 | フローラGLO | S-female6 | ザンマックス |

割付責任者は割付に従った試験食品を被験者番号毎に用意して、試験責任医師に送付する。その際、試験食品の入った容器には被験者番号のみを記載する。これにより、割付責任者のみが被験者に割り当てられた試験食品の種類を知っているが、試験責任医師、試験分担医師、眼科検査員、被験者は内容を知らないことになる。

## 4.7．試験食品

以下の2製剤とする。

①ザンマックス　ルテインサプリメント：

１粒中にルテイン10mg、ゼアキサンチン1mgを含有する濃褐色のソフトカプセル剤。なお、ルテインはサフラワーオイル懸濁製品である。

株式会社光洋商会が提供を行う。

②フローラGLO　ルテインサプリメント：

１粒中にルテイン（FloraGLO®、DSM株式会社）10mg、ゼアキサンチン0.05mgを含有する濃褐色のソフトカプセル剤。なお、ルテインはサフラワーオイル懸濁製品である。

## 4.8．サプリメントの用法･用量

　上記のいずれかを1日１回1粒を朝食後に飲料水と共に服用する。ただし、朝食時に飲み忘れた場合は同日中ならいつでもよいこととする。

## 4.9．服用期間

　6ヶ月間とする。

**5．中止基準とその手順**

5.1．中止基準

以下に記す患者個々人における中止基準、または試験の一部および全体の中止基準に該当する場合は、試験を中止する。

なお、下記の中止基準は、患者の安全性の確保、および倫理的観点から設定した。

5.2．患者個々人における中止基準

1. 被験者が同意を撤回した場合、中止を希望した場合
2. 試験開始後に被験者の都合で予定していた検査・観察の実施が不可能であることが判明した場合
3. 被験者の都合により試験が中断された場合（転居、多忙、追跡不能等）
4. 試験開始後に有害事象が発現し継続が困難であると試験責任医師が判断した場合
5. 試験開始後、被験者が対象症例ではないことが判明した場合
6. 被験者が試験担当医師の指示どおり摂取していないことが判明した場合
7. 偶発的な事故が発生した場合
8. その他、試験責任医師師が試験対象として不適格と判断した場合

5.3．試験の一部および試験全体の中止基準

1)重篤な有害事象の発現等により試験の継続が困難であり、中止が妥当と試験実施責任者が判断した場合

2)試験実施医療機関の長から中止の指示があった場合

5.4．中止後の対応

1)有害事象の発現の為中止した場合には、理由の如何に関わらず試験責任医師は適切な処置を講ずる。

2)試験責任医師は、理由不明で来院しなくなった中止例について、最終投与終了予定日から4週以内に必ず患者に連絡をとり、来院を促し、転帰調査を実施する。

**9．評価**

9.1．主要評価項目

黄斑色素密度の変化

9.2．副次的評価項目

①遠見、近見視力変化

②コントラスト・グレア感度変化

③黄斑ERGの変化

④血漿ルテイン濃度の変化

# **10．臨床研究に参加することにより予想される利益及び不利益**

　ルテインサプリメント内服により血漿ルテイン濃度と黄斑色素量が増加することが期待できる。

　使用されるサプリメントは両者とも市販品であり、重篤な副作用は報告されていない。摂取量も市販品の使用説明書どおりの量である。したがって、服用による不利益はないと予想される。ただし、個体により何らかの胃腸障害（便秘など）を訴える場合は予想される。

　黄斑色素測定時には散瞳を行うので、散瞳剤アレルギーによる充血・羞明などの症状発現の可能性がある。また、散瞳後は数時間にわたって調節力が低下する。

　採血に伴う痛みがある。

　当該サプリメントは提供会社より会社負担にて支給される。また、すべての眼科的検査、血漿濃度測定費用は眼科部門運営費から支出される。したがって、被検者の経済的負担はない。

# **11．研究終了後の対応**

　試験責任医師は、試験終了後、速やかに医療機関の長に試験の終了報告書を提出する。

なお、研究終了後の被験者への対応については、個々に決定する。

# **12．安全面への配慮**

被験者が服用するサプリメントに含有されているルテイン、ゼアキサンチンはもともと食物に含まれている栄養成分である。今回の服用量「１日10mg」はRitcherらが行った試験での投与量と同じで、副作用は報告されていない。

両サプリメントは国内での製造・販売許可を得て市販している製品と同じ成分である。これらの製品に関する副作用報告はこれまでのところみられない。

測定に使用される共鳴ラマン分光装置、眼底自家蛍光分光装置はともにヒトに使用する場合の安全性が立証されている^4^。また、これまで施行した両装置を使用する臨床試験でも障害の見られた例は皆無である。

# **13．倫理面への配慮**

本試験は、ヘルシンキ宣言に基づく倫理的原則および臨床研究に関する倫理指針（2003年7月30日厚生労働省）に従い、本試験実施計画書を遵守して実施する。

## 13.1．倫理委員会による審査・承認

　本試験は、あらかじめ医療機関の臨床試験審査委員会において本試験実施計画書等の内容、試験責任医師および試験分担医師の適格性等について審査を受ける。臨床試験審査委員会が試験の実施を承認した後に実施する。

　なお、試験期間を通じ、臨床試験審査委員会の審査の対象となる文書が追加、更新または改定された場合、（軽微な追加、更新または改定は除く）にも同様に審査を受けるものとする。

## 13.2．被験者の同意（インフォームド・コンセント）

　本試験の参加については、被検者の完全な自由意思による同意を得るものとする。試験担当医師は、本試験の実施にあたっては倫理的な配慮を慎重にし、下記の内容について十分説明した上で、文書にて被検者本人の同意を得る。同意文書には、説明を行った試験担当医師および同意した被験者が署名し、日付を記入し、同意書原本をカルテに保管し、コピーを１部被験者に渡すこととする。

## 13.3．被験者への新たな情報の提供

　本試験期間中に、被験者の同意に関連し得る新たな重要な情報あるいは本試験に継続して参加するかどうかについての被験者の意思に影響を与える可能性のある情報が得られた場合は、試験責任医師は速やかに当該情報に基づき同意説明文書等を改訂し、医療機関の臨床試験審査委員会の承認を得る。試験責任医師または試験分担医師は当該情報を速やかに被験者に説明し、新たに本試験への継続参加について自由意思による同意を文書により得、その同意文書の写しおよび改訂された説明文書を被験者に渡すものとする。

## 13.4．被験者のプライバシーの保護

　被験者のデータの取り扱い等については、被験者のプライバシーの保護に十分な配慮をする。すなわち、被験者の氏名やイニシャルは使用せず、被験者識別コードで特定するものとする。

# **14．研究の実施に伴う費用及び利害関係について**

　試験にまつわる諸検査、診察は算定しない。試験食品は無償提供を得る。

　光洋商会から病院あてに受託研究費の支給を受ける。

# **15．研究に伴う補償の有無**

　補償はありませんが、誠意をもって対応いたします。

被験者の篤志によるもので謝礼金は発生しない。ただし、6ヶ月間の試験完了例には光洋商会のルテインサプリメンと商品6か月分を支給する。

# **16．公表に関する取り決め**

　本試験実施計画書に基づいて行われた試験成績は聖隷浜松病院に帰属するものとする。成績の公表の際には被験者の秘密を保全する。

# **17．研究実施期間**

試験期間：　2010年7月1日　から　2011年3月31日

# **18．試験実施体制**

## 18.1．実施医療機関試験責任医師

## 聖隷浜松病院

〒430-8558 静岡県浜松市中区住吉2-12-12

TEL : 053-474-2222 FAX : 053-471-6050

## 18.2．試験責任医師

尾花　明

## 聖隷浜松病院眼科部長・浜松医科大学　光量子医学研究センター客員教授

## 18.3．臨床研究試験担当医師：

聖隷浜松病院　眼科医長　郷渡　有子

〒430-8558 静岡県浜松市住吉2-12-12

TEL : 053-474-2222 FAX : 053-471-6050

## 18.4．サプリメント提供会社：

株式会社光洋商会

〒103-0023　東京都中央区日本橋本町3-6-2　小津本館ビル8F

TEL : 03-3639-8555 FAX : 03-3667-9719

〒530-0002　大阪府大阪市北区曽根崎新地2-6-23　ＭＦ桜橋ビル10F

TEL 06-6341-3119 FAX 06-6348-1732

担当者　柴山信幸

KATRA PHYTOCHEM (INDIA) PRIVATE LIMITED

#1134, 1^st^ Floor, 100ft Road, HAL Ⅱ Stage, Bangalore-560 008, India

文献

1 Bernstein PS, Zhao DY, et al. Resonance raman measurement of macular carotenoids in normal subjects and in Age-related macular degeneration patients. Ophthalmology;109: 1780-87, 2002.

2 Ritcher S, Stiles W, et al. Double-masked, placebo-controlled, randomized trial of lutein and antioxidant supplementation in the intervention of atrophic age-related macular degeneration: the Veterans LAST study (Lutein Antioxidant Supplementation Trial). Optometry 75:216-30,2004.

3 Obana A, Hiramitsu T et al. Macular carotenoid levels of normal subjects and age-related maculopathy patients in a Japanese population. Ophthalmology, in print, 2007.

4 Sharifzadeh M, Bernstein PS, Gellermann W. Nonmydriatic fluorescence-based quantitative imaging of human macular pigment distributions. Opt.Soc.Am.A 23:2373-87, 2006.
