# Supplementary material for: Changes in Macular Pigment Optical Density and Serum Lutein Concentration in Japanese Subjects Taking Two Different Lutein Supplements
Source: PLoS One. 2015 Oct 9;10(10):e0139257. doi: 10.1371/journal.pone.0139257 (PMC4599964; doi:10.1371/journal.pone.0139257)
Supplement: S2 Protocol — (DOCX) [file pone.0139257.s012.docx]

**Clinical Study Protocol (Translated to English)**

**The effects of two types of lutein supplements on macular pigment and visual functions**

Supervisory investigator Akira Obana

Seirei Hamamatsu General Hospital

# 1. Introduction

## 1.1. Background

Human macula has yellow pigment called macular pigment that consists of three carotenoids, lutein ((3R,3’R,6’R)-lutein), zeaxanthin ((3R,3’R)-zeaxanthin), and *meso*-zeaxanthin ((3R,3’S;*meso*)-zeaxanthin).(Fig. 1)


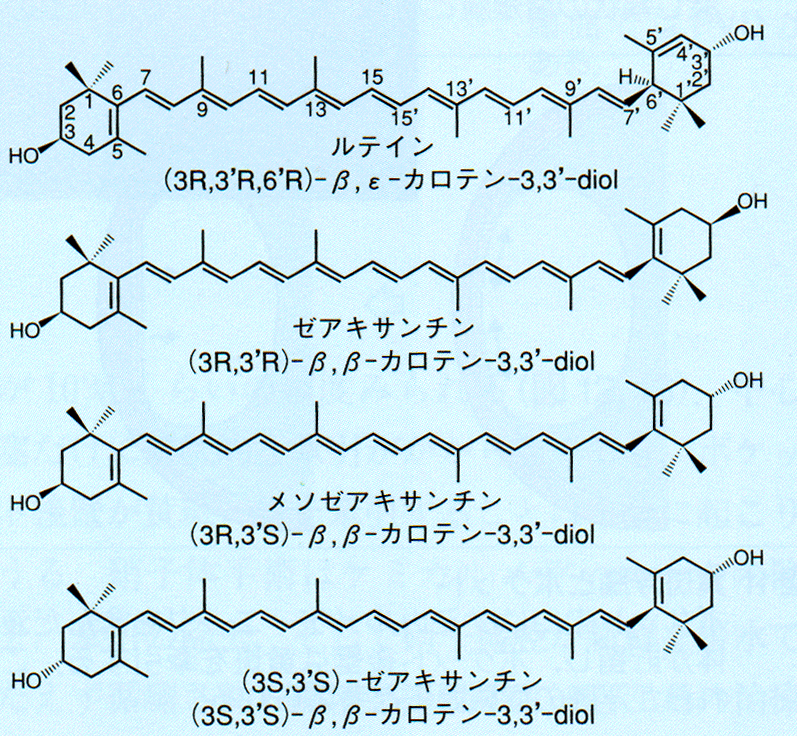
Fig.1 Structure of lutein and zeaxanthin

Humans are not able to synthesize lutein and zeaxanthin, and they are obtained from dietary sources such as green leafy vegetables and as supplements. Hydrophobic lutein and zeaxanthin are absorbed into the small intestine in the micellarized forms, and they are uptaken by the retinal pigment epithelial cells from the choroiocapillaris and stored in the inner plexiform layers of the retina. The uptake and accumulation of lutein and zeaxanthin in the retina is affected by many factors. Several receptor proteins participate in the absorption of lutein and zeaxanthin to be in the intestine and in their transport into the retina. Scavenger receptor class B type 1 (SR-B1) participates not only in the uptake of lutein and zeaxanthin absorption in the intestine, but it is also involved in the process in their transport from the choriocapillaris to the retinal pigment epithelium (RPE). Interphotoreceptor retinoid binding protein (IRBP) may work as a transporter from the RPE cells to the outer segment of the photoreceptor cells. Within the retina, lutein and zeaxanthin deposit specifically via their respective specific binding proteins (pi isoform of glutathione S-transferase (GSTP1) for lutein, steroidogenic acute regulatory domain 3(StARD3) for zeaxanthin. *Meso*-zeaxanthin is not contained in the foods and it is thought to be mostly converted from lutein in the retina.

Recently, researchers are studying the relation between the macular pigment and age-related macular degeneration (AMD). AMD is a leading cause of legal blindness in aged people in the US and Europe, and the number of patients with AMD is increasing even in Asian countries. AMD is divided into two types, atrophic AMD and exudative AMD. There is no effective therapy to atrophic AMD. Photodynamic therapy and intravitreous injection of anti-VEGF agents have been developed for the treatment of exudative AMD, but these treatments do not cure AMD and the treatment has to be repeated for years. Patients with AMD suffer from central scotoma and dismorphopsia and visual disturbance reduces the quality of life. AMD is a multifactorial disorder and genetic factors, aging, and various environmental factors are involved in its onset. Recently, the oxidant stress in the retina caused by the external light is attracting the attention as one of the environmental factors. External light is absorbed by the visual pigment in the photoreceptor cells to create the visual perception, and activates intraretinal oxygen and lipofuscin to produce singlet oxygen and radicals which cause oxidation of the photoreceptor cell membrane and injury of photoreceptor cells. Macular pigment absorbs blue light and acts as a filter that might attenuate photochemical damage on the retina from blue light. It also works as an antioxidant that may protect against light-induced oxidative damage in the retina by quenching oxygen radicals. These light protection effects of macular pigment have been expected to prevent AMD.

On the other hand, macular pigment has some effects on visual functions. The relationship between macular pigment and visual functions have been investigated and several reports suggested the improvement of contrast sensitivity, glare sensitivity and other functions such as photostress recovery and visual discomfort, although some studies failed to show the effectiveness.

We have reported in our previous studies that macular pigment optical density (MPOD) decreases with age, MPOD is lower in men than women, MPOD of the eyes with AMD is lower than healthy eyes and even the patient’s fellow eye free from AMD has lower MPOD than healthy eyes. We also revealed that MPOD and serum concentration of lutein increased under lutein intake (10 mg/day) in the persons not having AMD but did not in some patients with AMD. In these previous studies, we used FlorGLO as lutein supplements. The particle size of lutein affects bioabvailability. Generally, small size of lutein particle is thought to be solved into lipid and micellarized more efficiently than large size of particle, and high efficacy of micellarization of ingested carotenoids in the intestine had a marked effect on the extent to which the compounds are absorbed**.**

In the current study, we compared the efficacy to increase MPOD levels and plasma lutein concentration in normal, healthy Japanese subjects between two lutein supplements; one was FloraGLO lutein (Kemin Japan, Tokyo, Japan) that was supposed to be most widely used in the world and the other was Xanmax lutein (Katra Phytocem, Bangalore, India) that has smaller particle size than FlorGLO.

# 2. Study objectives

## 2.1. Study objectives

Assess the equivalence of two lutein supplements, FloraGLO and Xanmax on the efficacy of MPOD increase and plasma concentration of lutein in healthy subjects. Assess the improvement of visual functions with the increase of MPOD.

## **3. Subjects**

3.1. Subjects

Japanese

Healthy volunteers

Members or employees of Seirei Hamamatsu General Hospital

3.2. Inclusion criteria

No ocular pathologies detected by slit-lamp biomicroscopy and fundus ophthalmoscopy

Visual acuity of 0.8 or better at the time of the MPOD measurement

A spherical equivalent refractive error of less than -6.0 dioptor

No gastrointestinal diseases that could cause disturbance of dietary absorption

No diabetes

No history of lutein supplementation

No allergy to lutein and zeaxanthin

No history of smoking at least within one year

## 3.3. Exclusion criteria

Eye with cataract to the degree having substantial effects on the measurement of the macular pigment density

Eye of which mydriatic pupil diameter is less than 6.5 mm

Other subject assessed ineligible by the investigator

## 3.4. Number of subjects planned

Thirty-six patients in total (table 1)

Table 1 Number of subjects

|  | | Xanmax | | FlorGLO | | total |
| --- | --- | --- | --- | --- | --- | --- |
|  |  | M | F | M | F |  |
| Young age group | 20-34 yr | 3 | 3 | 3 | 3 | 12 |
| Middle age group | 35-49 yr | 3 | 3 | 3 | 3 | 12 |
| Senile age group | 50-64 yr | 3 | 3 | 3 | 3 | 12 |
| total | | 9 | 9 | 9 | 9 | 36 |

# 4. Study protocol

## 4.1. Study design

Randomized, double-blind, parallel-group comparison study in a single institute

## 4.2. Study procedures

Investigators perform history taking and ophthalmological examination ( measurement of visual acuity, measurement of intraocular pressure, fundoscopic and slit-lamp biomicroscopic observation under mydriasis) and evaluate whether the subjects admit the inclusion or exclusion criteria. Supervisory investigator determines the subject eyes.

All subjects received explanation on the detail of this study and signed an informed consent form. After obtaining written informed consent, subject number was assigned to each one in order to the enrollment.

The supervisory investigator will prepare and store the table covering the subject number, subject name, original medical record ID, date of consent, and date of start of administration.　(table 2).

Table 2 Subject records

|  | name | ID | Date of consent | Date of study start |  | name | ID | Date of consent | Date of study start |
| --- | --- | --- | --- | --- | --- | --- | --- | --- | --- |
| Y-male1 |  |  |  |  | Y-female1 |  |  |  |  |
| Y-male2 |  |  |  |  | Y-female2 |  |  |  |  |
| Y-male3 |  |  |  |  | Y-female3 |  |  |  |  |
| Y-male4 |  |  |  |  | Y-female4 |  |  |  |  |
| Y-male5 |  |  |  |  | Y-female5 |  |  |  |  |
| Y-male6 |  |  |  |  | Y-female6 |  |  |  |  |
|  | | | | | | | | | |
| M-male1 |  |  |  |  | M-female1 |  |  |  |  |
| M-male2 |  |  |  |  | M-female2 |  |  |  |  |
| M-male3 |  |  |  |  | M-female3 |  |  |  |  |
| M-male4 |  |  |  |  | M-female4 |  |  |  |  |
| M-male5 |  |  |  |  | M-female5 |  |  |  |  |
| M-male6 |  |  |  |  | M-female6 |  |  |  |  |
|  | | | | | | | | | |
| S-male1 |  |  |  |  | S-female1 |  |  |  |  |
| S-male2 |  |  |  |  | S-female2 |  |  |  |  |
| S-male3 |  |  |  |  | S-female3 |  |  |  |  |
| S-male4 |  |  |  |  | S-female4 |  |  |  |  |
| S-male5 |  |  |  |  | S-female5 |  |  |  |  |
| S-male6 |  |  |  |  | S-female6 |  |  |  |  |

### 4.3 Ophthalmological examinations

Measurement of far and near visual acuity, Measurement of contrast/glare sensitivity, Focal electroretinography (macular ERG), Optical coherence tomography (OCT), Color fundus photography, Measurement of MPOD

Measurement of MPOD

Make the subject eye mydriatic by the instillation of the mydriatic eye drop. Measure the pupil diameter. It is desirable that subjects have at least 7.0 mm of the mydriatic pupil diameter. Exclude the subject having the mydriatic pupil diameter of less than 6.5 mm.

Use the resonance Raman spectroscopy for the measurement. On each day of measurement, perform the pretest measurement using the standard device and retain the record.

Perform three repetitions of measurement per eye and retain all measurement data in the original medical record.

4.4 Measurement of the plasma lutein level

Obtain about 2 mL of venous blood using the heparin-added vacuum blood collection tube. Centrifuge the blood sample (4°C, 3000 rotations, 15 minutes) immediately and collect each exactly half of obtained plasma to two sample tubes. Freeze-store the plasma samples at the temperature of -20°C or under until they are submitted to the analysis center.

Diagnostic Division of Otsuka Pharmaceutical Co.,Ltd.

12F Shinagawa Grand Central Tower, 2-16-4 Minato-ku, Tokyo 108-8242

TEL: 03-6361-7304　 FAX: 03-6717-1467

4.5. Study schedule

Study schedule follows table 3.

## Table 3 Study schedule

|  | Explanations and obtainment of consent, enrollment (start of intake from the next day) | 1m | 2m | 3m | 4m | 5m | 6m |
| --- | --- | --- | --- | --- | --- | --- | --- |
| Visual acuity | ○ |  |  | ○ |  |  | ○ |
| Contrast・Glare sensitivity | ○ |  |  | ○ |  |  | ○ |
| Macular ERG | ○ |  |  |  |  |  | ○ |
| OCT | ○ |  |  |  |  |  | ○ |
| Fundus photography | ○ |  |  |  |  |  |  |
| Macular pigment measurement | ○ | ○ | ○ | ○ | ○ | ○ | ○ |
| Blood sample collection | ○ |  |  | ○ |  |  | ○ |

4.6. Assignment of the investigational supplement

The assignment personnel will prepare the table to assign the subjects with two supplements, FloraGLO and Xanmax corresponding to the subject number. The number of subjects assigned each supplement has to be equal between men and women. For example, in the group of young men, three men are assigned FloraGLO and three Xanmax.

Table 4. Example of allocation (The actual assignment is different from this)

|  | Investigational  food |  | Investigational  food |
| --- | --- | --- | --- |
| Y-male1 | Xanmax | Y-female1 | FloraGLO |
| Y-male2 | Xanmax | Y-female2 | Xanmax |
| Y-male3 | FloraGLO | Y-female3 | FloraGLO |
| Y-male4 | FloraGLO | Y-female4 | Xanmax |
| Y-male5 | Xanmax | Y-female5 | Xanmax |
| Y-male6 | FloraGLO | Y-female6 | FloraGLO |
|  |  |  |  |
| M-male1 | Xanmax | M-female1 | FloraGLO |
| M-male2 | FloraGLO | M-female2 | Xanmax |
| M-male3 | Xanmax | M-female3 | Xanmax |
| M-male4 | FloraGLO | M-female4 | FloraGLO |
| M-male5 | FloraGLO | M-female5 | FloraGLO |
| M-male6 | Xanmax | M-female6 | Xanmax |
|  |  |  |  |
| S-male1 | Xanmax | S-female1 | FloraGLO |
| S-male2 | Xanmax | S-female2 | Xanmax |
| S-male3 | Xanmax | S-female3 | FloraGLO |
| S-male4 | FloraGLO | S-female4 | Xanmax |
| S-male5 | FloraGLO | S-female5 | FloraGLO |
| S-male6 | FloraGLO | S-female6 | Xanmax |

The assignment personnel will prepare the investigational food according to the allocation table, and send them to the supervisory investigator. The subject number has to be written clearly on each investigational food. Thus, only assignment personnel knows the kind of investigational food assigned to each subject, and the supervisory investigator, investigators, examiners and subjects do not know the kind of investigational food.

# 4.7. Investigational food

1. Xanmax lutein:

One capsule contains 10 mg of lutein and 1mg of zeaxanthin. Suspension is safflower oil.

1. FloraGLO lutein (DSM Co.Ltd):

One capsule contains 10 mg of lutein and 0.05mg of zeaxanthin. Suspension is corn oil.

## 4.8. intake of supplement

The subjects will take one capsule once a day after meals with water according to the allocation.

4.9. Administration period

Six months

# 5. Discontinuation criteria and procedures

## 5.1. Discontinuation criteria

Discontinue the study in the individual subject if the subject meets the following subject-specific discontinuation criteria. Discontinue the whole or part of the study if the discontinuation criteria apply.

The following discontinuation criteria are determined to secure the safety of the subjects and from the ethical viewpoint.

### 5.2. Discontinuation criteria for individual subjects

1. If the subject withdraws the consent or requests the discontinuation
2. If the subject turns out not able to undergo the scheduled examinations/observations for the subject's own reason after the start of the study
3. If the study is suspended for the subject's own reason (e.g. transfer, change of doctor/hospital, business, inability of follow)
4. If the investigator assesses it difficult to continue the study because of the AE that has taken place after the start of the study
5. If the subject turns out to be ineligible after the start of the study
6. If the subject turns out not to have taken the investigational food as instructed by the investigator
7. In the event of onset of an incidental accident
8. If otherwise the investigator assesses the subject to be ineligible for the study

### 5.3. Whole or part of the study discontinuation criteria

1. If the investigator assesses it difficult to continue the study and that the study should be discontinued because of the onset of a serious AE
2. If the director of the study center instructs to discontinue the study

## 5.4 Procedures to follow after the discontinuation

1. Take measures necessary after the discontinuation. When the study is discontinued because of the AE, the investigator must take measures as appropriate irrespective of the reason.
2. For no show of unknown reason, the investigator will never fail to contact the subject within 4 weeks after the scheduled last administration to solicit the visit and investigate the outcome.

# 9. Evaluation (note; The number is mistaken in the original protocol written in Japanese)

## 9.1. Primary variable

Changes in the macular pigment optical density

### 9.2. Secondary variables

1. Changes in the far and near visual acuity
2. Changes in the contrast and glare sensitivity
3. Change in macular ERG
4. Changes in the plasma lutein level

# 10. Anticipated benefits and disbenefits of participation in the clinical study

Lutein intake is expected to improve the MPOD and plasma concentration of lutein. Lutein, which is a food ingredient, and no specific AE has been reported at the usual dose. The dose investigated is the usual dose of commercial products, therefore no AE is expected. It is speculated, however, some subjects complain gastrointestinal disorders such as constipation.

Measurement of MPOD is performed with mydriasis. There is a possibility of allergic reaction to mydriatic agents, and loss of accommodation for a few hours.

Taking blood sample is painful.

Investigational foods are supplied by supplement supplier. Study-related examinations and cost of measurement of the plasma lutein level will be covered by department of Ophthalmology, Seirei Hamamastu General Hospital. The subject will not incur any cost as a result of the participation in the study.

# 11. Procedures to follow after the study

The investigator will submit the study completion report to the director of the study center immediately after the completion of the study. Post-study procedures for individual subjects will be decided individually.

## **12** **Safety consideration**

Lutein and zeaxanthin contained in the capsule are nutritional ingredients of food. The dose of 10 mg/day is identical to the dose of previous study performed by Ritcher et al, and no adverse effects were reported.

Both supplements used in this study are same to products commercially available in Japan, and there have been no reports of adverse effects on these products.

The safety of Raman spectroscopy has been established for human usage. There were no adverse effects by using this device in our previous studies.

# 13. Ethical conduct of the study

The study is to be performed in accordance with the ethical principles based on the Declaration of Helsinki.

13.1. The study receives judgment on the study protocol and appropriateness of the investigators before the start of the study by the institutional review board. The study can start after the approval of the institutional review board.

## 13.2. Patient consent

The investigator will adequately explain the purpose and other details of the study using the explanatory and consent document approved in advance by IRB and supply the explanatory document to the subject prior to the start of the study. Obtain the subject's voluntary written consent to participate in the study. The subject and the investigator who has given the explanations will sign the consent document.

If the subject is assessed incapable of giving consent, disqualify the subject.

The investigator will provide the date of obtainment of consent in the CRF, have the subject sign the consent document in the relevant box unless otherwise agreed upon for the study center, supply the consent document to the subject, and submit the original of the signature page of the consent document to the IRB or other relevant department of the study center for storage.

After obtaining consent from the subject, the investigator or subinvestigator will allocate the subject number.

13.3. In the case of obtaining any information considered to affect the subject's willingness to continuously participate in the study, the investigator or subinvestigator will promptly supply the information to the subject, retain the record of information supply in the original medical record, and record the oral obtainment of re-consent. If any specific procedures are employed in the study center, retain the records according to the procedures. In the case of acknowledging the need to revise the explanatory and consent document based on the information, the investigator will discuss with the sponsor and make necessary revisions immediately. The investigator will submit the revised explanatory and consent document to the IRB by mediation of the director of the study center and gain approval. The investigator or subinvestigator will obtain the re-consent for the revised explanatory and consent document according to the procedures designated by the study center if any.

# 14. Study cost and interest

Study-related examinations and medical observations will not be assessed to health insurance. The investigational foods are supplied with free of charge. Seirei Hamamatsu General Hospital get grant from Koyo Mercantile Co., Ltd.

# 15. Availability of study-related compensation

The clinical study is to be performed based on the previous reports carefully according to the scientific protocol. If the subject has any ADR or health damage associated with the study during or after the completion of the study, the doctor will provide appropriate medical care and treatment.

The subject will not receive any remuneration. The subjects who completed the study receive commercial products of Koyo Mercantile Co., Ltd.

# 16. Agreement upon the release of data

The study data obtained in accordance with the study protocol belong to Seirei Hamamatsu General hospital. Release of the data will be discussed and decided by the supervisory investigator and all investigators. When the data are released, the privacy of the subjects will be kept confidential by anonymity.

# 17. Anticipated study period

July 1, 2010 to March 31, 2011

# 18. Study organization

## 18.1 Supervisory investigator

Akira Obana:

Director of Department of Ophthalmology, Seirei Hamamatsu General Hospital

Visisting professor, Photonmedical Research Center, Hamamatsu University School of Medicine

2-12-12 Sumiyoshi, Hamamatsu City, Shizuoka Prefecture 430-8558

TEL: 053-474-2222 FAX: 053-471-6050

## 18.2 Study investigators

Department of Ophthalmology, Seirei Hamamatsu General Hospital
Investigator: Akira Obana, Yuko Gohto

2-12-12 Sumiyoshi, Hamamatsu City, Shizuoka Prefecture 430-8558

TEL: 053-474-2222 FAX: 053-471-6050

## 18.3 Supplement supplier

Koyo Mercantile Co., Ltd

person in charge: Nobuyuki Shibayama

8F, Ozu-Honkan, 3-6-2 Nihonbashi Hontachi, Chuo-ku, Tokyo 103-0023

TEL: 03-3639-8555 FAX: 03-3667-9719

10F, MF Sakurabashi, 2-6-23 Sonezakishinti, kita-ku, Osaka 530-0002

<TEL:06-6341-3119> FAZ:06-6348-1732

KATRA PHYTOCHEM (INDIA) PRIVATE LIMITED

#1134, 1^st^ Floor, 100ft Road, HAL Ⅱ Stage, Bangalore-560 008, India

References

1 Bernstein PS, Zhao DY, et al. Resonance raman measurement of macular carotenoids in normal subjects and in Age-related macular degeneration patients. Ophthalmology;109: 1780-87, 2002.

2 Ritcher S, Stiles W, et al. Double-masked, placebo-controlled, randomized trial of lutein and antioxidant supplementation in the intervention of atrophic age-related macular degeneration: the Veterans LAST study (Lutein Antioxidant Supplementation Trial). Optometry 75:216-30,2004.

3 Obana A, Hiramitsu T et al. Macular carotenoid levels of normal subjects and age-related maculopathy patients in a Japanese population. Ophthalmology, in print, 2007.

4 Sharifzadeh M, Bernstein PS, Gellermann W. Nonmydriatic fluorescence-based quantitative imaging of human macular pigment distributions. Opt.Soc.Am.A 23:2373-87, 2006.
